# Molecular mapping of *qBK1*^*WD*^, a major QTL for bakanae disease resistance in rice

**DOI:** 10.1186/s12284-017-0197-7

**Published:** 2018-01-10

**Authors:** Sais-Beul Lee, Yeon-Jae Hur, Jun-Hyeon Cho, Jong-Hee Lee, Tae-Heon Kim, Soo-Min Cho, You-Chun Song, Young-Su Seo, Jungkwan Lee, Tae-sung Kim, Yong-Jin Park, Myung-Kyu Oh, Dong-Soo Park

**Affiliations:** 1 National Institute of Crop Science, Milyang, Miryang, 50424 Republic of Korea; 2 Department of Microbiology, Pusan National University, Pusan, 46241 Republic of Korea; 3 Department of Applied Biology, Dong-A University, Pusan, 49135 Republic of Korea; 4 Department of Agriculture, Korea National Open University, Seoul, 03087 Republic of Korea; 5 Department of Plant Resources, Kongju National University, Yesan, 32439 Republic of Korea

**Keywords:** Rice, Bakanae, *Gibberella fujikuroi*, QTL mapping, Resistance, Gene pyramiding

## Abstract

**Background:**

Bakanae or foot rot disease is a prominent disease of rice caused by *Gibberella fujikuroi*. This disease may infect rice plants from the pre-emergence stage to the mature stage. In recent years, raising rice seedlings in seed boxes for mechanical transplanting has increased the incidence of many seedling diseases; only a few rice varieties have been reported to exhibit resistance to bakanae disease. In this study, we attempted to identify quantitative trait loci (QTLs) conferring bakanae disease resistance from the highly resistant *japonica* variety Wonseadaesoo.

**Results:**

A primary QTL study using the genotypes/phenotypes of the recombinant inbred lines (RILs) indicated that the locus *qBK1*^*WD*^ conferring resistance to bakanae disease from Wonseadaesoo was located in a 1.59 Mb interval delimited on the physical map between chr01_13542347 (13.54 Mb) and chr01_15132528 (15.13 Mb). The log of odds (LOD) score of *qBK1*^*WD*^ was 8.29, accounting for 20.2% of the total phenotypic variation. We further identified a gene pyramiding effect of two QTLs, *qBK*^*WD*^ and previously developed *qBK1*. The mean proportion of healthy plant for 31 F_4_ RILs that had no resistance genes was 35.3%, which was similar to that of the susceptible check variety Ilpum. The proportion of healthy plants for the lines with only *qBK*^*WD*^ or *qBK1* was 66.1% and 55.5%, respectively, which was significantly higher than that of the lines without resistance genes and that of Ilpum. The mean proportion of the healthy plant for 15 F_4_ RILs harboring both *qBK*^*WD*^ and *qBK1* was 80.2%, which was significantly higher than that of the lines with only *qBK*^*WD*^ or *qBK1*.

**Conclusion:**

Introducing *qBK*^*WD*^ or pyramiding the QTLs *qBK*^*WD*^ and *qBK1* could provide effective tools for breeding rice with bakanae disease resistance. To our knowledge, this is the first report on a gene pyramiding effect that provides higher resistance against bakanae disease.

**Electronic supplementary material:**

The online version of this article (10.1186/s12284-017-0197-7) contains supplementary material, which is available to authorized users.

## Background

Bakanae disease is a disease of rice caused by *Gibberella fujikuroi* that was first described in Japan and now is widely distributed throughout Asia, Africa, North America, and Italy (Ou 1985; Prà et al. 2010). The common bakanae disease symptoms in rice plant are abnormal elongation such as tall, lanky tillers with pale green flag leaves, dried-up leaves, and infertile panicles (Mew and Gonzales 2002; Ou 1985). Bakanae disease decreases rice grain yield by 20–50% in Japan (Ou 1985) and 15–25% in India (Gupta et al. 2015). In Korea, 28.8% of the seedboxes for seedlings nursery were afected with bakanae disease in 2006 (Park et al. 2009), and 9.3% were affected in 2014. In recent years, raising rice seedlings in seedboxes for mechanical transplanting has coincided with many seedling disease problems that were not prevalent in open-field nurseries used for manual transplanting. Bakanae disease has become a serious problem in the breeding of hybrid rice, which involves increased use of seedbeds for plant growth (Li and Luo 1997; Yang et al. 2003). The most common management practice for bakanae is treating the seeds with hot water or fungicides. The hot water immersion method (Hayasaka et al. 2001) is ineffective for severely infected rice seeds because the hot water does not make contact with the pericarp of the rice seed. The application of fungicides cannot fully control fungal spores either, and fungicide resistant strains of bakanae have been reported (Ogawa 1988; Park et al. 2009; Kim et al. 2010; Lee et al. 2011). Cultivation of resistant cultivars potentially represents a cost-effective and environmentally friendly way to control this disease. However, extensive screening for bakanae resistant rice germplasm has identified only a few rice varieties (Li et al. 1993; Lv 1994; Khokhar and Jaffrey 2002; Kim et al. 2014).

It is necessary to identify resistance genes that can be used for marker-assisted selection in rice breeding and for understanding the mechanisms of resistance. Several quantitative trait loci (QTL) associated with bakanae disease resistance have been identified in previous studies. Yang et al. (2006) identified two QTLs on chromosome 1 and chromosome 10 by in vitro evaluation of the Chunjiang 06/TN1 doubled haploid population, which explained 13.4% and 13.3% of phenotypic variance. Hur et al. (2015) identified a major QTL, *qBK1*, from 168 BC_6_F_4_ near isogenic lines generated by crossing the resistant *indica* variety Shingwang with the susceptible *japonica* variety Ilpum. *qBK1* is located within a 520 kb region between simple sequence repeat (SSR) markers RM8144 (19.30 Mb) and RM11295 (23.72 Mb) based on the Nipponbare genome sequence. The RM9 marker showed the highest log of odds (LOD) score (33.21) and accounted for 65% of the phenotypic variation. Fiyaz et al. (2016) identified three QTLs, *qBK1.1*, *qBK1.2*, and *qBK1.3,* which accounted for 4.76%, 24.74%, and 6.49% of phenotypic variation, respectively. Varieties with a single resistance gene may lose their resistance by the emergence of new population of fungal isolates (Wang and Valent 2009). Four *Fusarium* species including *F. andiyazi*, *F. fujikuroi*, *F. proliferatum*, and *F. verticillioides* from the *G. fujikuroi* species complex have been reported to be associated with bakanae disease (Wulff et al. 2010). Hence, identifying new resistance genes from diverse sources is important for rice breeding programs to defend against bakanae disease by enhancing the resistance level and/or help to overcome the breakdown of resistance genes. In this study, we generated 200 recombinant inbred lines (RILs) from a cross between a resistant (Wonseadaesoo) and a susceptible (Junam) *japonica* variety using insertion/deletion (InDel) and tetra markers (Ye et al. 2001), which were developed based on resequencing of the two parental varieties, to identify new QTLs for bakanae disease resistance in rice. The results of this study are expected to provide useful information for developing resistant rice lines that contain single or multiple major QTLs by pyramiding the resistance genes for bakanae disease.

## Results

### Bakanae disease bioassay in parental rice varieties

**Fig. 1:**
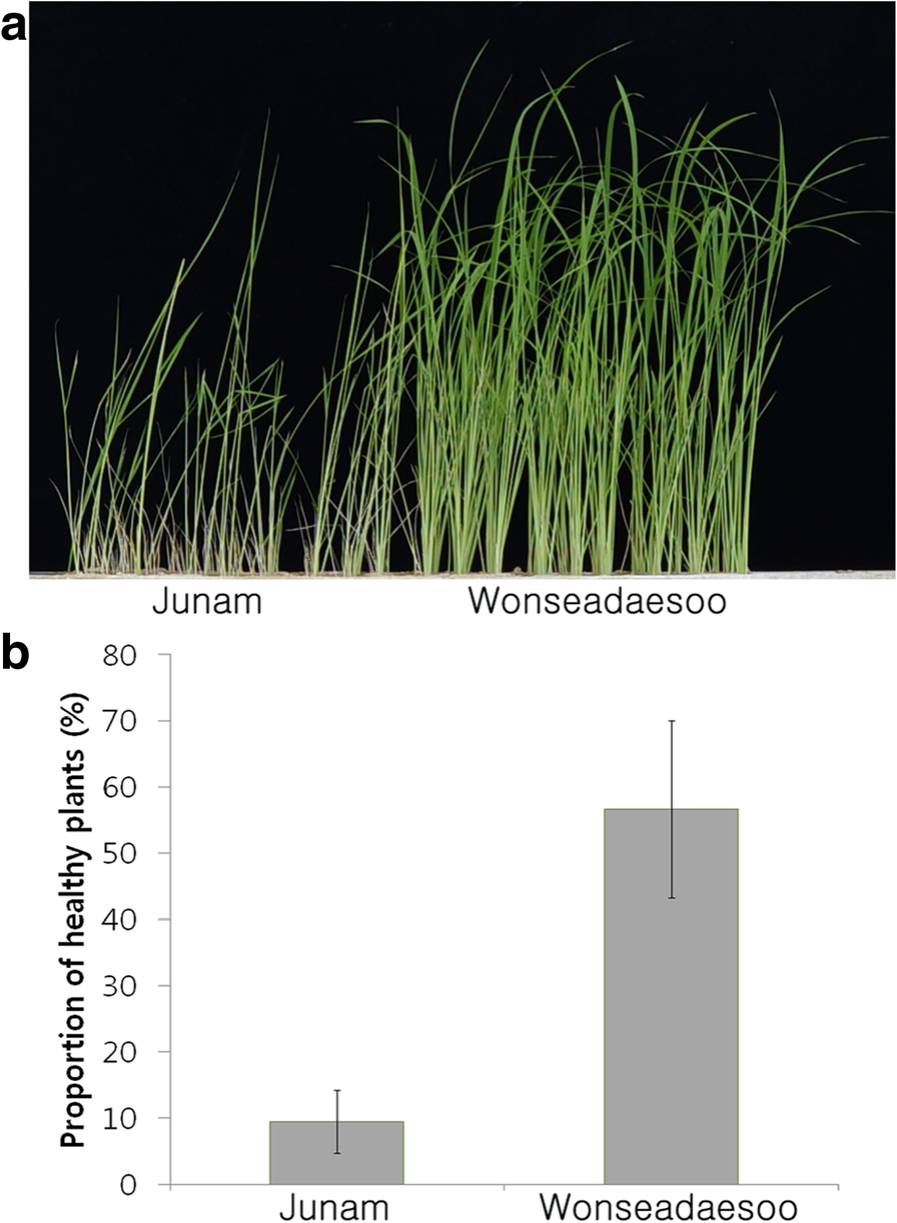
Phenotype (**a**) and proportion of healthy plants (**b**) in parental varieties infected with the *Fusarium fujikuroi* isolate CF283

To investigate the host resistance to bakanae disease, the proportion of healthy plants in Wonseadaesoo (resistant) and Junam (susceptible) were measured after inoculation with virulent *F. fujikuroi* isolate CF283 (Kim et al. 2014). Junam showed typical bakanae disease symptoms such as abnormal elongation, pale green leaves, or drying up of the whole plantlets as compared to Wonseadaesoo (Fig. 1a). The proportion of healthy Junam and Wonseadaesoo plants was 11.0% and 65.7%, respectively (Fig. 1b).

**Fig. 2:**
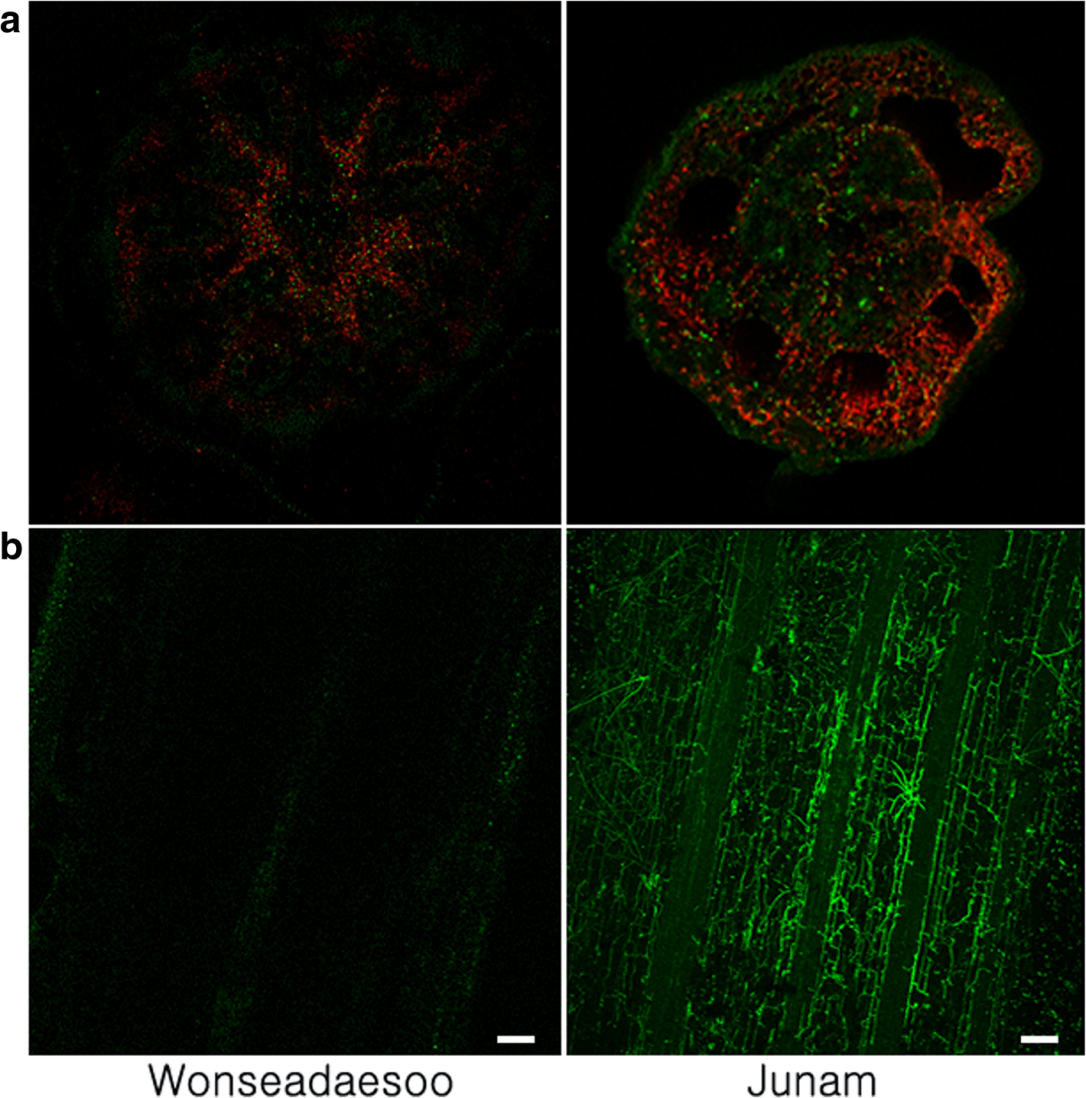
Confocal imaging of Wonseadaesoo and Junam rice plants infected with CF283GFP *Fusarium fujikuroi* isolates. **a** Radial and (**b**) longitudinal sections of the basal stem (Scale bar = 20 μm)

We generated green fluorescent protein (GFP)-tagged *F. fujikuroi* CF283 isolate and inoculated two rice varieties for microscopic observation. Ten days after inoculation, infected plants from each variety with typical disease symptoms were subjected to a confocal microscopy analysis. Confocal imaging of radial sections of the basal stem showed that the fungus penetrated the stele in both varieties, and was more abundant in the susceptible Junam variety than it was in the resistant Wonseadaesoo (Fig. 2).

### QTL analysis and mapping of bakanae disease resistance using 200 F_4_ RILs

**Fig. 3:**
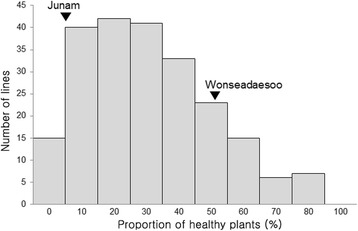
Frequency distribution of the proportion of healthy plants (*n* = 200) in 200 F_4_ recombinant inbred lines

A bioassay for bakanae disease was conducted with 200 F_4_ RILs derived from the cross Junam/Wonseadaesoo using the *F. fujikuroi* isolate CF283. The proportion of healthy plants for the 200 F_4_ RILs exhibited a continuous distribution (0–100%; Fig. 3). The average proportion of healthy Junam plants was 10.9% (0–12.5%; *n* = 11) and that of Wonseadaesoo was 56.7% (51.9–72.2%; *n* = 11).

A genotyping by sequencing (GBS) experiment detected 16,941 single-nucleotide polymorphism (SNP) and 1591 InDel loci between Junam and Wonseadaesoo (data not shown). Finally, 135 InDel markers that showed a polymorphism in 3% agarose gel were selected from 277 InDel markers designed from resequencing data and a polymorphism survey of the whole chromosomes of Junam and Wonseadaesoo. A genetic linkage map of Junam and Wonseadaesoo was constructed with 135 polymorphic markers that covered a total length of 2134 cM with an average interval of 15.8 cM (Additional file 1: Figure S1). Primary QTL mapping showed that a significant QTL associated with bakanae disease resistance at the seedling stage was located between the InDel markers, chr01_10336087 and chr01_26628298 on chromosome 1, and it was designated *qBK1*^*WD*^*.* The LOD score of *qBK1*^*WD*^ was 8.29, which accounted for 20.2% of the total phenotypic variation (Table 1). The location of *qBK1*^*WD*^ was narrowed down by analyzing the chromosome segment introgression lines in the region detected from primary mapping. The *qBK1*^*WD*^ region from the primary mapping was further analyzed with 46 additional InDel markers for the insertion/deletion sites and 15 tetra markers for the SNPs based on the resequencing data of the Wonseadaesoo and Junam varieties. Seven InDel markers and three tetra markers were selected as polymorphic markers between the parents to narrow down the position of the *qBK1*^*WD*^ region (Additional file 2: Table S1 and Table S2).

**Table 1:** Quantitative trait loci (QTLs) detected by composite interval mapping for bakanae disease resistance

QTL	Chromosome	Left marker	Right marker	LOD	PVE (%)	Additive effect
*qBK* ^*WD*^	1	chr01_10336087	chr01_26628298	8.29	20.2	−9.53

*LOD* log of odds score, *PVE* percentage of phenotypic variation explained

**Fig. 4:**
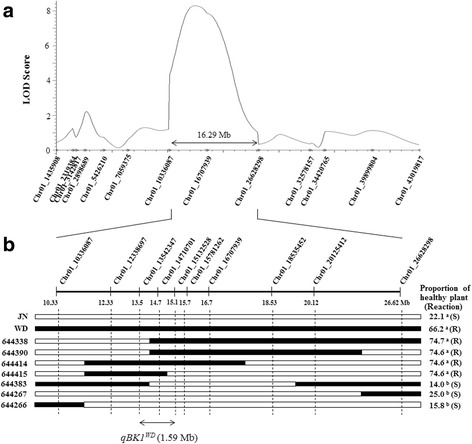
Quantitative trait locus (QTL) analysis of *qBK1*^*WD*^ using recombinant inbred lines (RILs) derived from a cross between Wonseadaesoo and Junam rice plants. **a** In primary mapping, *qBK1*^*WD*^ was identified in a 16.29 Mb region between the InDel markers chr01_10336087 and chr01_26628298 on chromosome 11. **b** Location of *qBK1*^*WD*^ was narrowed down to 1.59 Mb by finer mapping using homozygous recombinants. The proportion of healthy plant were calculated from biological replications. Values (%) of the proportion of healthy plant with different letters are significantly different by Duncan’s multiple range test at *P* = 0.05

**Fig. 5:**
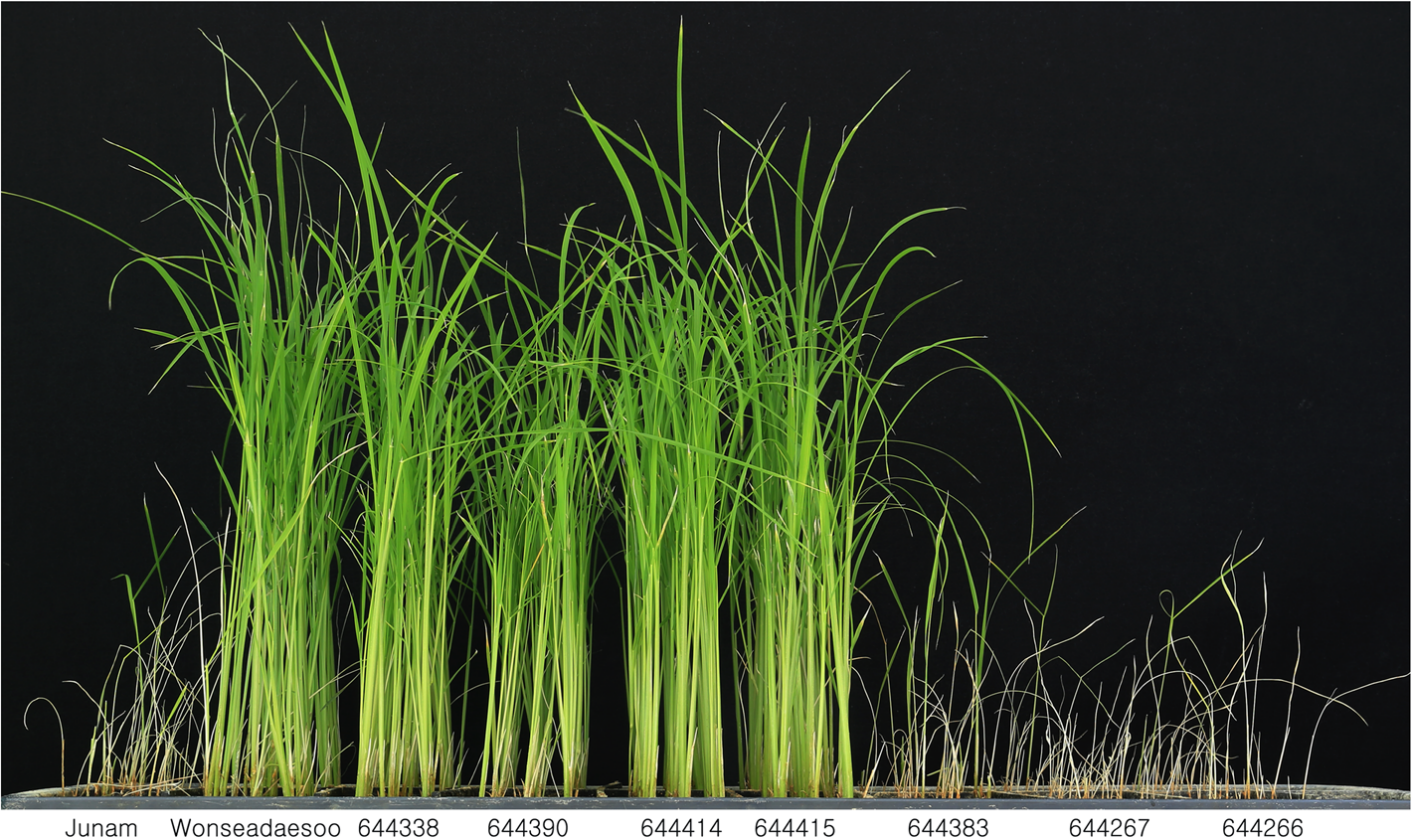
Phenotypic responses to bakanae disease in seven homozygous recombinants for finer mapping

Finally, seven homozygous reczombinants were selected from the F_4:5_ lines using 10 markers in the 16.29 Mb region between the InDel markers chr01_10336087 and chr01_2662898 (Figs. 4 and 5). The phenotypes of the recombinant lines indicated that the locus conferring resistance to *qBK1*^*WD*^ was approximately a 1.59 Mb interval delimited on the physical map between chr01_13542347 (13.54 Mb) and chr01_15132528 (15.13 Mb).

### Gene pyramiding effect of two bakanae disease resistance QTLs, *qBK*^*WD*^ and *qBK1*

To identify the pyramiding effect of the two major QTLs *qBK*^*WD*^ and *qBK1,* we generated 314 F_4_ RILs from a cross between Wonseadaesoo (harboring *qBK*^*WD*^) and YR24982–9-1 (harboring *qBK1*), and further selected 231 F_4_ lines showing homogeneous genotype with the molecular marker chr01_15132528 for *qBK*^*WD*^ (in this study) and RM9 for *qBK1* (Hur et al. 2015). The mean proportion of healthy plant for the F_4_ RILs plants with no resistance gene (aabb, 31 lines) was 35.3% (Table 2), which was similar to that of the susceptible control variety Ilpum (the mean of the 22 replicates was 39.2%; data not shown). The mean proportion of healthy plant in the lines with only *qBK*^*WD*^ (aaBB, 93 lines) and *qBK1* (AAbb, 92 lines) was 66.1% and 55.5%, respectively, which was significantly higher than that of the lines that had no resistance gene (aabb) or that of Ilpum. The mean proportion of healthy plant in the lines with both *qBK*^*WD*^ and *qBK1* (AABB, 15 lines) was 80.2%, which was significantly higher than that of the lines with a single QTL, *qBK*^*WD*^ (aaBB) or *qBK1* (AAbb).

**Table 2:** Mean proportion of healthy plants harboring individual gene combinations infected with *Fusarium fujikuroi* isolate CF283

Gene combination	AABB (*qBK1 + qBK*^*WD*^)	aaBB (*qBK*^*WD*^)	AAbb (*qBK1*)	aabb	Total
No. of lines	15	93	92	31	231
Mean proportion of healthy plants	80.2^a^	66.1^b^	55.5^c^	35.3^d^	59.3

Means with different characters of superscripts (a, b, c and d) indicate significant differences at *P* = 0.05

## Discussion

### Identification of *qBK*^*WD*^, a major QTL against bakanae disease resistance

Rice varieties with a single resistance gene are at an increased risk of being overcome by new virulent strains. Therefore, it is essential to improve various genetic resources against bakanae disease in rice breeding programs. In our previous study (Hur et al. 2016), the *japonica* germplasm Wonsaedaesoo showed the highest level of resistance (ratio of healthy plants was 97%) among the 254 rice germplasm accessions examined, and it showed a higher level of resistance than that of another bakanae-resistant plant Shingwang (ratio of healthy plants was 77%). In this study, we have attempted to identify new resistance loci from Wonseadaesoo to improve bakanae disease resistance in rice breeding programs. Our GBS results detected 16,941 SNP and 1571 InDel loci between Wonseadaesoo and Junam. In the primary mapping, *qBK*^*WD*^ was detected in the 16.29 Mb region between the physical positions 10.33 and 26.62 Mb on chromosome 1.

We further selected four additional InDel markers and three tetra markers to narrow down the position of the *qBK1*^*WD*^ region. Tetra-primer amplification has been described as an efficient and low-cost method for SNP genotyping (Ye et al. 2001, Chiapparino et al. 2004). This method uses two locus-specific outer primers that asymmetrically flank the SNP under investigation and two allele-specific inner primers. The different product sizes produced by one inner and outer primer pairs can be easily detected by polyacrylamide or agarose gel electrophoresis. Finer mapping revealed that the QTL *qBK*^*WD*^ is located in an approximately 1.59 Mb long interval between 13.54 and 15.13 Mb on chromosome 1 (Fig. 4).

Five QTLs related to bakanae disease resistance were previously identified on chromosome 1. Hur et al. (2015) identified *qBK1*, a major QTL from the Korean *indica* variety Shingwang. The *qB1*, identified by Yang et al. (2006), is located between RM7180 and RM486 (approximately 34 Mb long region). The *qBK1* (Hur et al. 2015) is located between RM8144 and RM11295 at the physical position of 23.2 and 23.72 Mb; this region is shared with *qBK1.1,* located between RM9 and RM11282 (Fiyaz et al. 2016). Both *qBK1.2* and *qBK1.3* are located between RM10153 and RM5336, which correspond to the physical positions of 3.10 and 3.36 Mb, and from RM10271 to RM35 at the physical position of 4.65 Mb regions (Fiyaz et al. 2016). Therefore, *qBK*^*WD*^ is a novel QTL for bakanae disease resistance, and which does not overlap with any of *qB1* (Yang et al. 2006), *qBK1* (Hur et al. 2015), *qBK1.1 qBK1.2*, or *qBK1.3* (Fiyaz et al. 2016).

### Application of marker-assisted selection (MAS) for the pyramiding of QTLs, *qBK*^*WD*^ + *qBK1*

Gene pyramiding through conventional phenotype assays in breeding crops is considered to be difficult and often impossible due to the dominance and epistatic effects of genes governing disease resistance and the limitations related to all year-round screening (Sundaram et al. 2009). Major QTLs for bakanae disease resistance were transferred into *japonica* rice by backcross breeding and marker-assisted selection, which would thus reduce the amount of time and labor-intensive bioassays required for the backcross procedure. Pyramiding multiple resistant genes in a single plant might confer higher and/or durable resistance against bakanae disease. The effects of pyramiding resistance genes have been investigated for several plant-microbe interactions. Pyramiding three bacterial blight resistance genes resulted in a high level of resistance and was expected to provide durable pathogen resistance (Singh et al. 2001, Pradhan et al. 2015). In other cases, pyramiding of resistant genes resulted in a level of resistance that was comparable to or even lower than that by a single gene. For example, Yasuda et al. (2015) reported that disease suppression in rice lines carrying pairs of resistance genes against rice blast is comparable to that observed in lines containing the gene with a stronger suppressive effect. Moreover, the number of lesions and the percentage of diseased leaf area in lines with several gene combinations such as *pi21 + Pi34* and *pi21 + Pi35* were significantly lower than those in lines with individual resistance genes. Therefore, investigating the effect of pyramiding genes involved in bakanae disease resistance is important to enhance the level of resistance in rice.

In our previous study of bakanae disease resistance, *qBK1* was mapped between the flanking markers RM8144 and RM11295; the RM9 marker had the highest LOD score and was therefore selected for marker-assisted foreground selection of *qBK1* into elite breeding materials to acquire bakanae disease resistance (Hur et al. 2015). The results presented herein revealed that rice lines harboring *qBK*^*WD*^ showed a higher level of resistance than those with *qBK1.* Furthermore, the pyramided rice lines harboring *qBK*^*WD*^ + *qBK1* had a much higher level of resistance than those harboring either *qBK*^*WD*^ or *qBK1*.

The development of a rice variety with a higher level of resistance against bakanae disease is a major challenge in many countries (Cumagun et al. 2011, Bashyal et al. 2014, Fiyaz et al. 2016, Hur et al. 2015). The study clearly establishes the utility of MAS in gene pyramiding such as that of the two-gene combination *qBK*^*WD*^ + *qBK1*, which can achieve higher resistance in many bakanae disease prone rice growing areas.

## Conclusions

Introducing *qBK*^*WD*^ and pyramiding the QTLs *qBK*^*WD*^ and *qBK1*, along with the utilization of the MAS could provide tools for the breeding of rice varieties with bakanae disease resistance. Further fine mapping studies will be needed to determine the actual candidate gene of the *qBK*^*WD*^ QTL by using additional molecular markers and recombinants.

## Methods

### Plant materials

Two rice varieties, the bakanae disease resistant *japonica* variety Wonseadaesoo and the susceptible *japonica* variety Junam, were used in this study. Wonseadaesoo was selected as the foremost resistant rice germplasm from 500 varieties screened with a newly developed fast and reproducible inoculation method for accurate evaluation of rice bakanae disease resistance (Kim et al. 2014; Hur et al. 2016). We generated 200 F_2:4_ RILs from a cross between Wonseadaesoo and Junam for QTL analysis. To identify the pyramiding effect of the two major QTLs *qBK*^*WD*^ and *qBK1*, 314 F_4_ RILs were further generated from a cross between Wonseadaesoo and YR24982–9-1 harboring *qBK*^*WD*^ and *qBK1*, respectively. YR24982–9-1 is a BC_5_F_5_ near isogenic line carrying *qBK1* selected from backcross lines between Shingwang as the donor, and Ilpum as the recurrent parent (Hur et al. 2015).

### Evaluation of bakanae resistance

The evaluation of bakanae disease was performed using a method modified from that described by Kim et al. (2014) and Hur et al. (2015, 2016). Our previous study revealed that 11 varieties including Wonseadaesoo were resistant to bakanae disease among 254 rice germplasm accessions examined for infection with *F. fujikuroi* isolate CF283 (Hur et al. 2016). The *F. fujikuroi* isolate CF283 was inoculated in potato dextrose broth (PDB) and cultured at 26 °C under continuous light for one week. The fungal spore concentration was adjusted to 1 × 10^6^ spores/mL with a hemocytometer to obtain standardized inoculums. Forty seeds per line were placed into a tissue-embedding cassette (M512, Simport, Beloeil, QC, Canada). The seeds in the tissue-embedding cassette were then surface sterilized in a hot water bath (57 °C) for 13 min and allowed to drain before they were soaked in a conidial suspension in another tray for 3 d at 26 °C with gentle shaking four times a day. After inoculation, 30 seeds per line were sown in nursery bed soil in a seedling tray. The inoculated seedlings were grown in a greenhouse at 28 ± 5 °C during the day and 23 ± 3 °C at night, in a 12 h light/dark cycle. The response to bakanae disease was evaluated by calculating the proportion of healthy plants in a given plot one month after sowing. Healthy and unhealthy plants were classified by the method described by Kim et al. (2014). Plants with the same phenotype as untreated plants or slight elongated seedlings with no thinness or yellowish coloring after infection were regarded as healthy plants. This method is fast, reproducible, and accurate in evaluating bakanae disease resistance in rice when compared to methods using direct indicator of bakanae disease resistance such as shoot elongation after GA treatment (Kim et al. 2014).

### Generation of transgenic *F. fujikuroi* strains carrying green fluorescent protein (GFP)

To investigate the infection process of *F. fujikuroi* in rice, GFP was introduced into the CF283 isolate as previously described (Lee et al. 2008). In brief, a DNA fragment (3.4 kb) of a cassette including GFP and hygromycin resistance genes was amplified from the pIGPAPA plasmid (Horwitz et al., 1999), which carries the gene encoding GFP fused to the *Neurospora crassa* isocitrate lyase promoter and *hygB* fused to the *Aspergillus nidulans TrpC* promoter, with primers ICL-F1 (5′-GGGCCCCACACGGACTCAAAC-3′) and HYG-F1 (5′-GGCTTGGCTGGAGCTAGTGGAGG-3′). The fragment was directly introduced into CF283 protoplasts with a polyethylene glycol-mediated method (Lee et al. 2002). Transformed CF283GFP constitutively expressing GFP had similar pathogenicity, growth, conidiation, and pigmentation to that of untransformed CF283 isolate.

### Microscopy

The disease symptoms of 10-day-old plants infected with CF283GFP isolate were observed by confocal microscopy. Symptomatic tissues were analyzed using a Zeiss 510 laser scanning confocal microscope (Carl Zeiss Microscopy GmbH, Jena, Germany). The GFP was excited with a 488 nm excitation line and detected with a BP 500–530 IR emission filter.

### Selection of InDel markers from sequencing data

The number of polymorphic SSR markers was not sufficient for *japonica*/*japonica* mapping populations. Only four out of 100 SSR markers from the Gramene database (http://www.gramene.org) showed polymorphic differences between Wonseadaesoo and Junam varieties in our preliminary test for marker survey. We re-sequenced Wonseadaesoo and Junam by GBS, and selected 135 polymorphic InDel markers that could differentiate the genotypes of Wonseadaesoo and Junam using agarose gel electrophoresis from 11,393 InDel loci between the two varieties covering the 12 rice chromosomes. The PCR cycling conditions for InDel markers were 2 min at 94 °C, followed by 35 cycles at 94 °C for 20 s, 55 to 60 °C for 30 s, and 72 °C for 40 s, and a final extension at 72 °C for 7 min. The PCR cycling conditions for tetra primers were as follows: 5 min at 94 °C, followed by 35 cycles at 94 °C for 30 s, 60 to 65 °C for 40 s, and 72 °C for 1 min, and a final extension at 72 °C for 7 min. The amplified products were separated using a 3% agarose gel electrophoresis and visualized with ethidium bromide.

### QTL and statistical analysis

The 200 F_2:4_ RILs that were derived from a cross between Wonseadaesoo and Junam were used for QTL analysis. The QTL analysis was performed by composite interval mapping with QTL IciMapping v4.0.6.0 software (Meng et al. 2015). LOD threshold of 7.2 was used to confirm the presence of putative SSR markers associated with bakanae disease resistance. The percentage of trait variation explained by a QTL and the additive effects were also estimated by QTL IciMapping program (Meng et al. 2015). Statistical differences between means were analyzed using Duncan’s multiple range test after one-way analysis of variance (ANOVA). The level of significance was designated at *P* < 0.05 and determined using the SAS 9.4 program (SAS Institute Inc., Cary, NC, USA).

## Additional files

Additional file 1: Figure S1. Linkage map constructed with 200 F_4_ recombinant inbred lines (RILs) derived from a cross between Wonseadaesoo and Junam rice plants (TIFF 4376 kb)

Additional file 2: Table S1. InDel markers used for the fine mapping of *qBK*^*WD*^. **Table S2.** Tetra markers used for the fine mapping of *qBK*^*WD*^ (DOC 36 kb)

## Abbreviations

GBS: genotyping by sequencing
GFP: green fluorescent protein
InDel: insertion/deletion
LOD: log of odds
MAS: marker-assisted selection
PDB: potato dextrose broth
QTLs: quantitative trait loci
RILs: recombinant inbred lines
SSR: simple sequence repeat
