# Supplementary material for: Molecular mapping of qBK1WD, a major QTL for bakanae disease resistance in rice
Source: Rice (N Y). 2018 Jan 10;11:3. doi: 10.1186/s12284-017-0197-7 (PMC5762613; doi:10.1186/s12284-017-0197-7)
Supplement: Supplementary file 2 — InDel markers used for the fine mapping of qBKWD. Table S2. Tetra markers used for the fine mapping of qBKWD (DOC 36 kb) [file 12284_2017_197_MOESM2_ESM.doc]

Table S1. InDel markers used for fine mapping of *qBK^WD^*

| Primer ID | Forward primer (5’-3’) | Reverse primer (5’-3’) |
| --- | --- | --- |
| Chr01_ 10336087 | TCTGGTTGGCCCAAATGAC | CACCTTCTTAGGAGGGCGAT |
| Chr01_ 12338697 | TGAAAACTTTCAACCCAGATTT | TTCGCACACCTCTTTACAAATG |
| Chr01_ 15781262 | CGTGGCTGCTCTTTTGATGT | GCGAAACACCTCCATGGTTA |
| Chr01_ 16707939 | AGCACATGCATCTTCCTAAACC | GCACAGCAAGGGCATAAACA |
| Chr01_ 18535452 | CATTCGACCATCGGTTTTGA | TGTGGTCTTTTCTTTGCCCA |
| Chr01_ 20125412 | TCTTTTAAAGTACTCCCTCC | TTTTAGTCTCACCAAGTTTG |
| Chr01_ 26628298 | AAAAATTTCCCTAATTACTCGTCA | AAACATGACACGCTATAGAAAACA |

*Table S2. Tetra markers used for fine mapping of qBK*WD

| Primer ID | Outer/Inner | Forward primer (5’-3’) | Reverse primer (5’-3’) |
| --- | --- | --- | --- |
| Chr01_ 13542347 | Outer | TCACCGAAGACAACGAGTGGTGACCACA | CCCATTGCCAAGTGGAGGTAAAGCTCGA |
|  | Inner | GTCTGATCCTCGTAGGGGATGATGCATGG | ATGGCTCTCATCGTGGTTGGGCTGGT |
| Chr01_ 14710701 | Outer | AGTTAAGTCCAAGCTGGAGTGCCGCGAT | CAACTCATTTTCGCCCCAGCCCTTGTAG |
|  | Inner | CTTTCGGCACACCCACATCCTTCACAAT | GCTCTTGTCGCCGTACCTTGTGGGTAATG |
| Chr01_ 15132528 | Outer | TCCCTCCATCCAAAATTATAAGGCCTAT | GGCAATGTAAGATGTAGAATGTGGCAGT |
|  | Inner | CAAGTTTGATTTTCCTTAAAAAGAGGAAGC | AAAACTCCCTCCGTCCCAAATTATATGA |
